# S92 phosphorylation induces structural changes in the N-terminus domain of human mitochondrial calcium uniporter

**DOI:** 10.1038/s41598-020-65994-y

**Published:** 2020-06-04

**Authors:** Youngjin Lee, Jongseo Park, Gihwan Lee, Sanghwa Yoon, Choon Kee Min, Tae Gyun Kim, Takenori Yamamoto, Do Han Kim, Keun Woo Lee, Soo Hyun Eom

**Affiliations:** 1 School of Life Sciences, Gwangju Institute of Science and Technology (GIST), Buk-gu, Gwangju 61005 Republic of Korea; 2 Steitz Center for Structural Biology, Gwangju Institute of Science and Technology (GIST), Buk-gu, Gwangju 61005 Republic of Korea; 3 Systems Biology Research Center, Gwangju Institute of Science and Technology (GIST), Buk-gu, Gwangju 61005 Republic of Korea; 4 Infection and Immunity Research Laboratory, Metabolic Regulation Research Center, Korea Research Institute of Bioscience and Biotechnology (KRIBB), Daejeon, 34141 Republic of Korea; 5 Division of Life Science, Division of Applied Life Science (BK21 Plus), Plant Molecular Biology and Biotechnology Research Center (PMBBRC), Research Institute of Natural Science (RINS), Gyeongsang National University (GNU), 501 Jinju-daero, Jinju, 52828 Republic of Korea; 6 Institute for Genome Research, Tokushima University, Kuramotocho-3, Tokushima, 770-8503 Japan; 7 Faculty of Pharmaceutical Sciences, Tokushima University, Shomachi-1, Tokushima, 770-8505 Japan; 8 Present Address: Molecular Cryo-Electron Microscopy Unit, Okinawa Institute of Science and Technology Graduate University, 1919-1 Tancha, Onna, Kunigami 904-0495 Japan

**Keywords:** X-ray crystallography, Biochemistry

## Abstract

The mitochondrial calcium uniporter (MCU) plays essential roles in mitochondrial calcium homeostasis and regulates cellular functions, such as energy synthesis, cell growth, and development. Thus, MCU activity is tightly controlled by its regulators as well as post-translational modification, including phosphorylation by protein kinases such as proline-rich tyrosine kinase 2 (Pyk2) and AMP-activated protein kinase (AMPK). In our *in vitro* kinase assay, the MCU N-terminal domain (NTD) was phosphorylated by protein kinase C isoforms (PKC_βII_, PKC_δ_, and PKC_ε_) localized in the mitochondrial matrix. In addition, we found the conserved S92 was phosphorylated by the PKC isoforms. To reveal the structural effect of MCU S92 phosphorylation (S92p), we determined crystal structures of the MCU NTD of S92E and D119A mutants and analysed the molecular dynamics simulation of WT and S92p. We observed conformational changes of the conserved loop2-loop4 (L2-L4 loops) in MCU NTD_S92E_, NTD_D119A_, and NTD_S92p_ due to the breakage of the S92-D119 hydrogen bond. The results suggest that the phosphorylation of S92 induces conformational changes as well as enhancements of the negative charges at the L2-L4 loops, which may affect the dimerization of two MCU-EMRE tetramers.

## Introduction

Under physiological conditions, mitochondria, which uptake and sequester Ca^2+^ into the matrix, play essential roles in the regulation of ATP synthesis through the tricarboxylic acid cycle (TCA), buffering of cytosolic Ca^2+^, and cell growth and development^1^. However, prolonged overload of mitochondrial Ca^2+^ uptake can trigger the production of large amounts of reactive oxygen species (ROS), induce opening of the mitochondrial permeability transition pore, cause disruption of mitochondrial membrane potential, and eventually lead to apoptotic and necrotic cell death^1^. The malfunction of mitochondrial Ca^2+^ homeostasis causes pathological diseases, including ischemia reperfusion, myocardial infarction, and epilepsy^2–4^.

A key pathway for mitochondrial Ca^2+^ uptake across the inner mitochondrial membrane (IMM) is through the mitochondrial calcium uniporter (MCU) complex, which facilitates Ca^2+^ entry into the IMM through the electrochemical potential gradient driven by the mitochondrial membrane potential (Δψ = approximately −180 mV)^5,6^. The MCU is the pore-forming subunit of the MCU complex and acts as a selective Ca^2+^ channel. The MCU complex consists of MCU, along with its regulatory proteins, MCU paralog (MCUb), mitochondrial calcium uptake 1, 2 & 3 (MICU1, MICU2, and MICU3), essential MCU regulator (EMRE), and mitochondrial calcium uniporter regulator 1 (MCUR1). The interaction of these regulatory proteins with MCU controls MCU Ca^2+^ uptake activity under different mitochondrial conditions and in different cell types^2,7,8^.

Mitochondrial calcium uptake greatly varies depending on the cell types and tissues due to the differential expression levels of MCU complex components^9,10^. The mitochondrial Ca^2+^ conductance measurements using whole-mitoplast current recording suggest that the level of Ca^2+^ entry mediated by MCU differs between mouse tissues, including skeletal muscle, heart, brown fat, kidney, and liver^9^. In addition, uncoupling proteins 2 and 3 (UCP2 and UCP3) interact with methylated MICU1 and can differentially modulate the mitochondrial calcium uptake in different cells, including HeLa, Ea.hy926, HUVEC, and PAEC cells^10^.

The role of the MCU as an essential Ca^2+^ channel for mitochondrial Ca^2+^ uptake is supported by functional studies of the MCU in human cells and mouse models^7,11–16^. Inhibition of Ca^2+^ uptake in the matrix has been previously demonstrated by blocking the MCU pore with ruthenium red (Ru360)^16^, and by genetic ablation of MCU^7,11^. Previous research has also shown that the microRNA miR-25 can reduce the mRNA level of MCU and directly down-regulate MCU expression, thus inhibiting the mitochondrial Ca^2+^ uptake^12^. In the MCU pore-forming region, mutations of negatively charged acidic residues (E257, D261, E264) in human MCU, have also been shown to inhibit the MCU activity^16^. Although the MCU knock-out mouse was reported as the mild phenotype, unexpected compensatory changes that affect cytosolic Ca^2+^ homeostasis or modulate mitochondrial Ca^2+^-dependent metabolism impair the short-term mitochondrial Ca^2+^ uptake at a “fight-or-flight” response^13–15^.

The N-terminal domain (NTD) of the MCU (MCU NTD) plays essential roles in the dimerization of two MCU-EMRE tetramers, MCUR1 interaction, MCUb NTD interaction, Mg^2+^ binding selectivity, redox sensor, and regulation of MCU Ca^2+^ uptake activity^6,7,17–19^. In addition, the MCU NTD can be altered by post-translational modifications in the mitochondrial matrix space^7,20,21^. Under inflammatory and hypoxic conditions, MCU undergoes S-glutathionylation in a highly conserved C97 residue and functions as a mitochondrial ROS sensor in the mitochondrial matrix^7^. Phosphorylation of the MCU (predicted Y158 in the NTD and Y289, Y317 in the C-terminal domain) by proline-rich tyrosine kinase 2 (Pyk2) induces an increase in the mitochondrial Ca^2+^ uptake by facilitating formation of the MCU channel via MCU oligomerization^20^. S57 phosphorylation in the MCU by AMP-activated protein kinase (AMPK) facilitates mitochondrial Ca^2+^ entry during mitosis and boosts mitochondrial respiration to maintain energy homeostasis^21^. Regulatory functions of MCU Ca^2+^ uptake by Ca^2+^/calmodulin-dependent protein kinase II (CaMKII) still remain controversial^22–26^, although Nguyen *et al*. suggest S92 phosphorylation (S92p) of MCU by CaMKII *in vivo*^25^. Since our study focuses on the S92p of MCU by protein kinase C (PKC), we exclude an argument about the functional role of MCU driven by CaMKII.

PKC isoforms, a heterogeneous family of serine/threonine (Ser/Thr) kinases, are encoded by nine genes (α, β, γ, δ, ε, η, θ, ι, ζ) in human^27^, and PKC_βII_ (one of the splice variants of PKC_β_), PKC_δ_, and PKC_ε_ are localized in the mitochondrial matrix in response to ROS^27–29^. PKC directly phosphorylates a wide range of cellular substrates and regulates various cellular functions, such as cell migration, differentiation, proliferation, senescence, and apoptosis^27–29^.

In this study, we observed that the MCU S92 was phosphorylated by mitochondrial PKC isoforms, including PKC_βII_, PKC_δ_, and PKC_ε_, via *in vitro* kinase assay. To uncover the structural effects of phosphorylated S92 (S92p), we determined two crystal structures of MCU NTD_S92E_, an S92p mimic, and NTD_D119A_ mutants at a resolution of 2.50 Å and 2.85 Å, respectively, and analysed the molecular dynamics simulation for NTD_WT_ and NTD_S92p_. We propose that phosphorylation at S92 induces conformational and electrostatic changes in the L2-L4 loops of the MCU NTD_WT_ due to the breakage of S92-D119 hydrogen bonds. As a result, it may affect the dimerization of the two MCU-EMRE tetramers.

## Results

### The MCU NTD S92 is phosphorylated by PKC_βII_, PKC_δ_, and PKC_ε_

**Figure 1:**
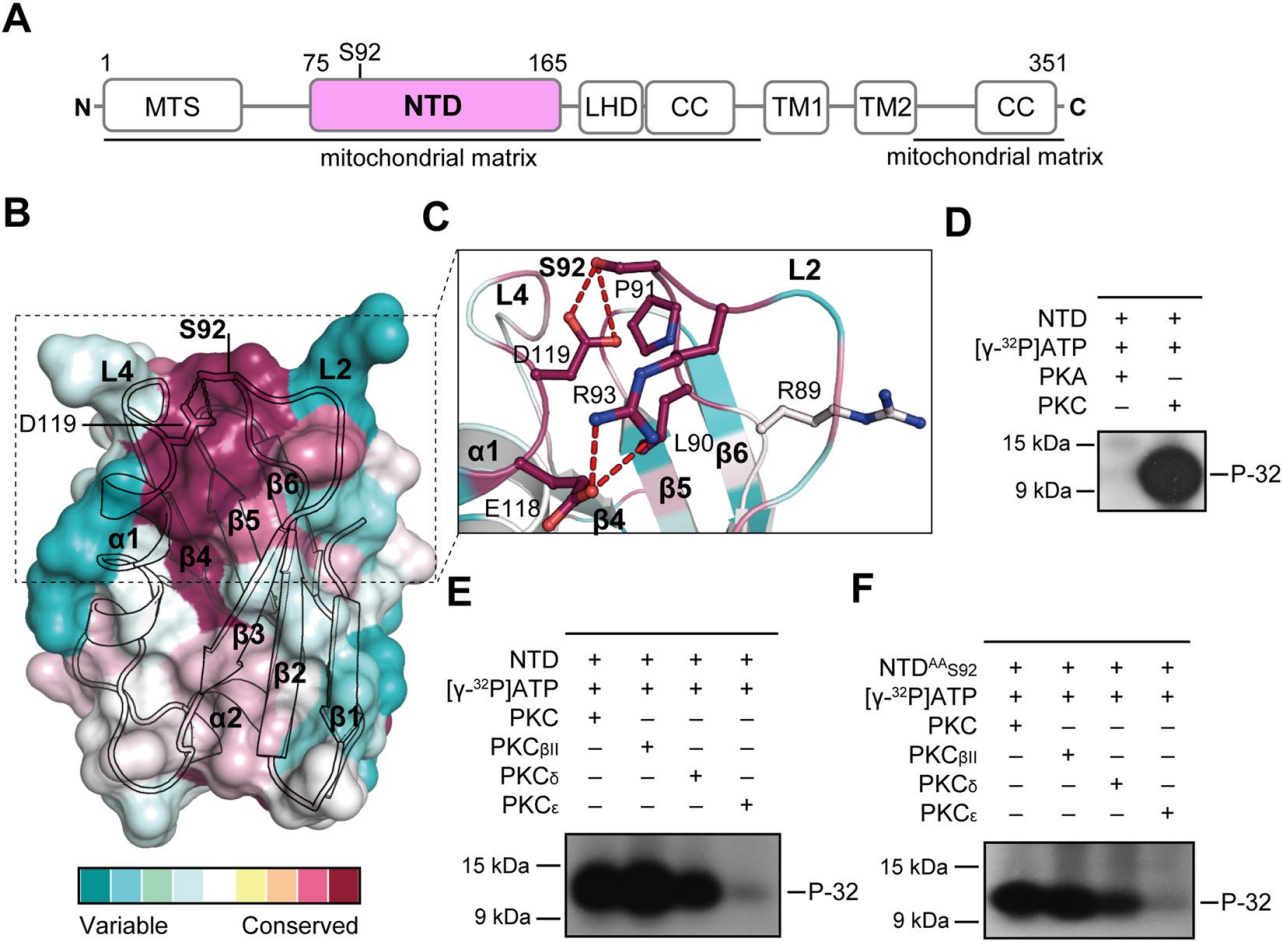
Conserved S92 is phosphorylated by protein kinase C isoforms. (**A**) Schematic diagram of the MCU. The MCU consists of a mitochondrial targeting sequence (MTS), N-terminal domain (NTD), linker helix domain (LHD), two transmembrane domains (TM1 and TM2), a TM linker (L), and two coiled-coils (CC). **(B)** Surface and ribbon diagrams of the MCU NTD coloured by scoring the residue conservation from 230 MCU NTD homologues using the ConSurf server. Highly conserved and variable residues are shown in red and green, respectively. The β-strands (β1 − β6), α-helices (α1, α2), and loops (L1 − L8) are shown in arrows, cylinders, and lines, respectively. **(C)** Detailed view of the highly conserved L2-L4 loop regions in the MCU NTD (PDB ID, 4XTB). The residues and hydrogen bonds are denoted in stick and dashed lines (red). **(D–F)** *In vitro* kinase assays of MCU NTD_WT_ (residues 75–165) (**D**,**E**) and MCU NTD^AA^_S92_ (**F**). Autoradiography analysis of MCU NTD_WT_ (residues 75–165) and MCU NTD^AA^_S92_ proteins that were incubated with protein kinase A (PKA), protein kinase C (PKC) isoforms (PKC mixture of α, β, and γ isoforms with lesser δ and ζ; PKC_βII_; PKC_δ_; PKC_ε_), and [γ-^32^P]ATP (P-32). We designed all Ser/Thr (T76, S87, S92, T100, S105, S107, S129, S138, T139, and T157) mutants of the MCU NTD except the S92 (MCU NTD^AA^_S92_). Full autoradiography results in Supplementary Fig S4. The reaction samples were resolved by SDS-PAGE, and visualized by autoradiography. Data are representative of three independent experiments.

The MCU NTD sequence, which is encoded by exon 3 and 4 (residues 75−165) of the *MCU* gene, was highly conserved based on 230 MCU NTD homologous protein sequences in the ConSurf server (Fig. 1A–C, Supplementary Fig. S1)^30^. The MCU NTD has six serines (S87, S92, S105, S107, S129, and S138) and four threonines (T76, T100, T139, and T157). Among these, the highly conserved S92 in the 89-RLPS-92 sequences (the RxxS motif, where x is any residue) was determined to be a putative recognition site for phosphorylation by Ser/Thr kinases containing CaMKII, cAMP-dependent protein kinases (PKA), and PKC, using the KinasePhos 2.0 server and Group-based Prediction System (GPS) 2.0 softwares (Fig. 1C, Supplementary Fig. S1)^22,31–35^. Previously, Nguyen *et al*. isolated mitochondria from vascular smooth muscle cells (VSMC) and detected the S92p of MCU by CaMKII using specific MCU S92p antibodies in an immunoblotting^25^. To further investigate whether S92 is phosphorylated by other Ser/Thr kinases such as PKA and PKC localized in the mitochondrial matrix^28,36,37^, we performed *in vitro* kinase assays with myelin basic protein (MBP; positive control), MCU NTD_WT_, MCU NTD^AA^_S92_ (all alanine mutations of the nine Ser/Thr residues in the NTD except S92), and [γ-^32^P]ATP. In control experiments, MBP, a multiple phosphorylation target by Ser/Thr kinases^38–40^, was phosphorylated by PKA and PKC isoforms (α, β, γ mixtures, βII, δ, and ε) (Supplementary Fig. S2). Under the same conditions, MCU NTD_WT_ was phosphorylated by PKC, but not by PKA (Fig. 1D) and MCU NTD^AA^_S92_ was also phosphorylated by PKC (Fig. 1F). In all nine PKC isoforms, three PKC isoforms, including PKC_βII_, PKC_δ_, and PKC_ε_ are localized in the mitochondrial matrix and regulate the reactive oxygen species (ROS) formation in the matrix^27–29^. In additional *in vitro* kinase assays, we observed that PKC_βII_, PKC_δ_, and PKC_ε_ phosphorylated S92, and that S92 phosphorylation activities by PKC_βII_ and PKC_δ_ were stronger than that of PKC_ε_ (Fig. 1E,F). Thus, we suggest that PKC_βII_, PKC_δ_, and PKC_ε_ localized in mitochondrial matrix can phosphorylate the S92 in the MCU NTD.

### In details of conformational and electrostatic changes of MCU NTD by S92 phosphorylation

**Figure 2:**
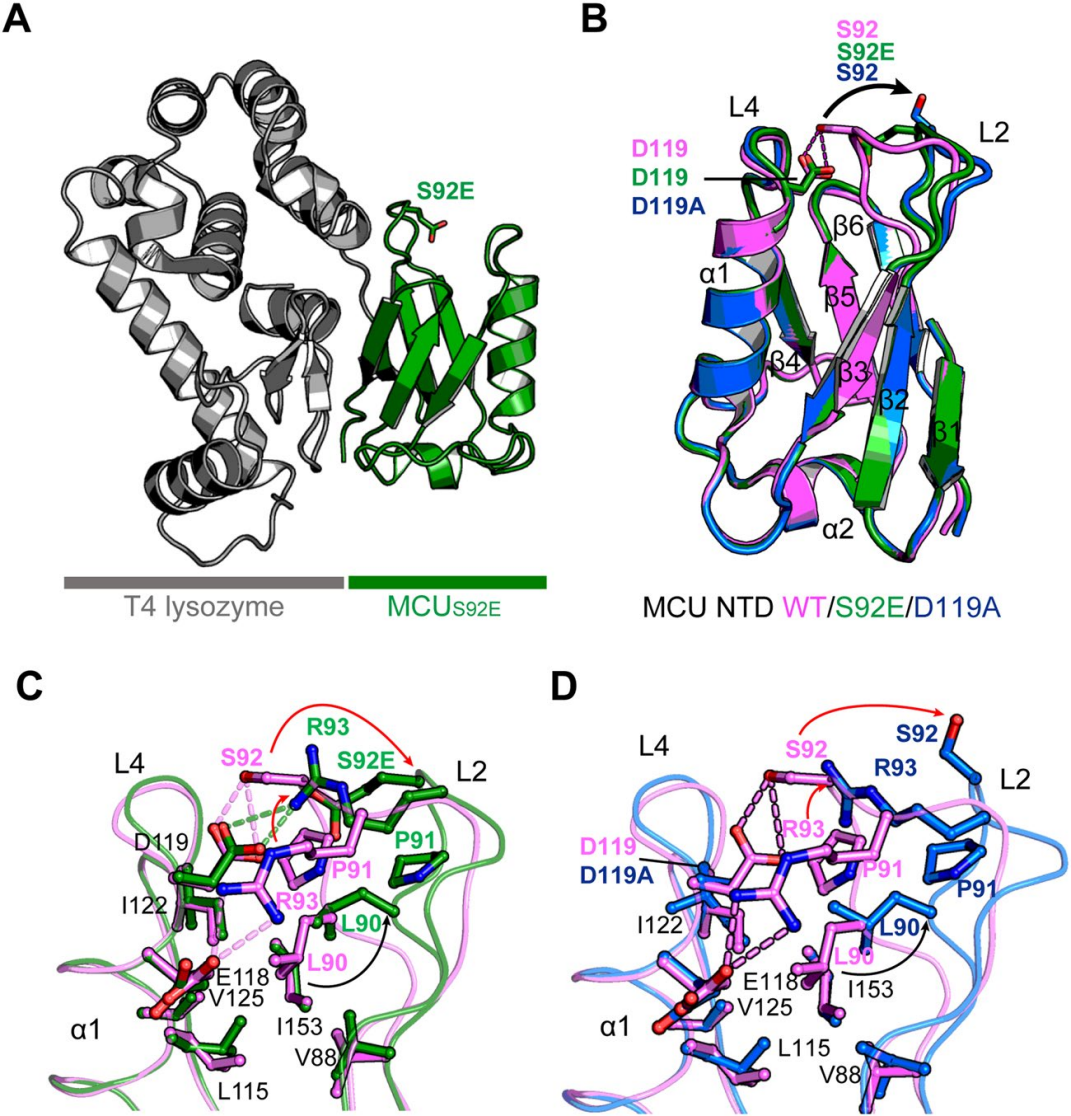
Structural comparison between the MCU NTD_WT_ and the NTD_S92E_ or NTD_D119A_ mutants. **(A)** Overall structure of MCU NTD_S92E_ mutant (green) fused with bacteriophage T4 lysozyme (gray) at the N-terminus end of the MCU NTD. **(B)** Ribbon diagrams of superimposed MCU NTD_WT_ (PDB ID: 4XSJ) and mutant structures of MCU NTD_S92E_ and MCU NTD_D119A_. The ribbon diagrams are represented in different colours: magenta (WT), green (S92E), and blue (D119A). The side chains of residues are shown in stick, the red dashed lines denote hydrogen bonds, and the L2 loop of conformational changes are represented by black arrows. **(C,D)** Detailed view of superimposed L2-L4 loops of MCU NTD_WT_ (magenta) and MCU NTD_S92E_ (green) (**C**) or MCU NTD_WT_ (magenta) and MCU NTD_D119A_ (blue) (**D**). The backbone and side chains of residues are represented in ribbon and stick, respectively. The arrows represent movement of the residues participating in hydrophobic interaction (black) or hydrogen bonds (red). Dashed-lines (green in S92E and magenta in WT) denote hydrogen bonds.

To reveal the structural effect of S92 phosphorylation in the MCU NTD, we generated the S92E mutant, an S92p mimic, of MCU NTD fused with the bacteriophage T4 lysozyme at the N-terminal end of MCU NTD (T4-MCU NTD_S92E_) to improve protein solubility for crystallographic studies^6^. We determined the structure of T4-MCU NTD_S92E_ at a resolution of 2.50 Å by molecular replacement using the MCU NTD_WT_ (PDB ID: 4XSJ) and T4 lysozyme (PDB ID: 2LZM) structures as templates (Fig. 2A; Table 1).

**Table 1:** Data collection and refinement statistics.

Proteins	T4 lysozyme-MCU NTD S92E	T4 lysozyme-MCU NTD D119A
PDB ID:	6JG0	6KVX
**Data Collection**		
Space group	*P*6_5_	*P*6_5_
X-ray source^a^	PAL-5C	PAL-5C
Detector	ADSC Q315	ADSC Q315
Wavelength (Å)	0.9795	0.9795
Unit cell: *a*, *b*, *c* (Å)	97.9, 97.9, 61.5	97.9, 97.9, 61.6
Resolution range (Å)^b^	50.0‒2.50 (2.54‒2.50)	50.0‒2.85 (2.90‒2.85)
*R*_merge_^c^	8.3 (61.9)	9.3 (59.1)
CC_1/2_^d^ in outer shell (%)	60.7	70.8
*I*/σ(*I)*	14.3 (3.0)	12.3 (3.0)
Completeness (%)	99.7 (99.8)	98.7 (99.8)
Redundancy	4.8 (3.5)	4.2 (4.1)
**Refinement**		
Resolution range (Å)	42.5‒2.50	29.0‒2.85
No. reflections	11122	7489
*R*_work_^e^ (%)/*R*_free_ (%)	19.0/26.0	18.0/26.2
**No. atoms/residues**		
Protein	2011/253	2027/253
SO_4_^2−^	10/2	20/4
Water	77	−
B-factors (Å^2^)		
Protein	52.1	55.0
SO_4_^2−^	88.6	81.9
Water	46.4	−
**Model statistics**		
rmsd bond length (Å)	0.012	0.014
rmsd bond angles (°)	1.40	1.20
Ramachandran plot (%) favoured/allowed/disallowed	97.6/2.4/0.0	96.0/4.0/0.0

^a^Beamline 5 C at Pohang Acceleratory Laboratory (PAL) in South Korea.

^b^Values in parentheses are for highest-resolution shell.

^c^*R*_merge_ = ∑_*h*_ ∑_*i*_ │I(*h*)_*i*_ − ‹I(*h*)›│/∑_*h*_ ∑_*i*_I(*h*)_*i*_, where I(*h*) is the intensity of reflection of *h*, ∑_*h*_ is the sum overall reflections and ∑_*i*_ is the sum over *i* measurements of reflection *h*.

^d^CC_1/2_ in outer shell were calculated from HKL2000.

^e^*R*_work_ = Σ_*hkl*_ ||F_*o*_ | -|F_*c*_ ||/Σ_*hkl*_ | F_*o*_ | ; 5% of the reflections were excluded for the *R*_free_ calculation.

The overall structure of MCU NTD_S92E_ was similar to the structure of MCU NTD_WT_ (PDB ID: 4XSJ) with root-mean-square deviation (RMSD) of 0.57 Å for 87 C_α_ atoms, and consisted of two helices (α1 and α2) and six β-strands (Fig. 2A,B). The S92-D119 in the L2-L4 loops of MCU NTD_WT_ formed a hydrogen bond at a distance of 2.5 Å; the R93 interacted with the E118 to form a salt bridge and stabilized the closed form of L2 loop (Fig. 2B,C). The mutation of S92 to E92 results in atomic clashes of the side chains between E92 and D119, broke the S92-D119 hydrogen bond, and induced conformational changes from the closed form of the L2 loop in MCU NTD_WT_ to the open form (Fig. 2B,C). The peptide backbone of the L2 loop in the MCU NTD_S92E_ moved away from L2 loop of MCU NTD_WT_ (C_α_ atom distance of 4.6 Å), and the side chain of R93 moved up to the position of S92 and formed a new hydrogen bond with E92 and D119. The MCU NTD_S92E_ L90 in the hydrophobic interior, which also contained V88, L115, I122, V125, and I153 in MCU NTD_S92E_ moved away from that of MCU NTD_WT_ at a distance of 2.2 Å (Fig. 2B,C). Based on the MCU NTD_S92E_ structure, we can suggest that the additional phosphate group by the S92 phosphorylation break the S92-D119 hydrogen bond due to atomic clashes between the phosphate group of S92p and the carboxyl group of D119, and induces a conformational change similar to that of the MCU NTD_S92E_.

In our previous studies, we unintentionally observed that the S92A mutation abolish the S92-D119 hydrogen bond in the structure of the MCU NTD_S92A_^6^ (Supplementary Fig. S3A). Intriguingly, the conformational changes of the L2-L4 loops in MCU NTD_S92A_ were similar to that of MCU NTD_S92E_ (Supplementary Fig. S3B) and were maintained in the open form of L2 loop in comparison with the closed form of MCU NTD_WT_ (Fig. 2C, Supplementary Fig. S3A), hypothesizing that S92 phosphorylation might modulate the open or closed conformation of the NTD L2-L4 loops.

To further investigate whether the S92-D119 hydrogen bond is important for maintaining the closed conformation of the L2-L4 loops, we prepared the D119A mutant to break the S92-D119 hydrogen bond. We determined the structure of the MCU NTD_D119A_ mutant fused with N-terminus T4-lysozyme fusion (T4-MCU NTD_D119A_) at 2.85 Å resolution. Overall, the structures of MCU NTD_WT_ and the MCU NTD_D119A_ mutant were similar, with an RMSD of 0.61 Å for 86 C_α_ atoms (Fig. 2B). As expected, the MCU NTD_D119A_ also broke the S92-D119 hydrogen bond from the L2-L4 loops of MCU NTD_WT_ and caused structural changes in the L2-L4 loops, similar to that observed in the structure of MCU NTD_S92E_ (Fig. 2B,D). The L2 loop conformation of the MCU NTD_D119A_ moved away at a C_α_ atom distance of 5.1 Å from that of MCU NTD_WT_, while the side chain of R93 residue, which moved up to the position of S92, did not form a new hydrogen bond because of lack of a hydrogen bonding counterpart by D119A mutation (Fig. 2B,D).

**Figure 3:**
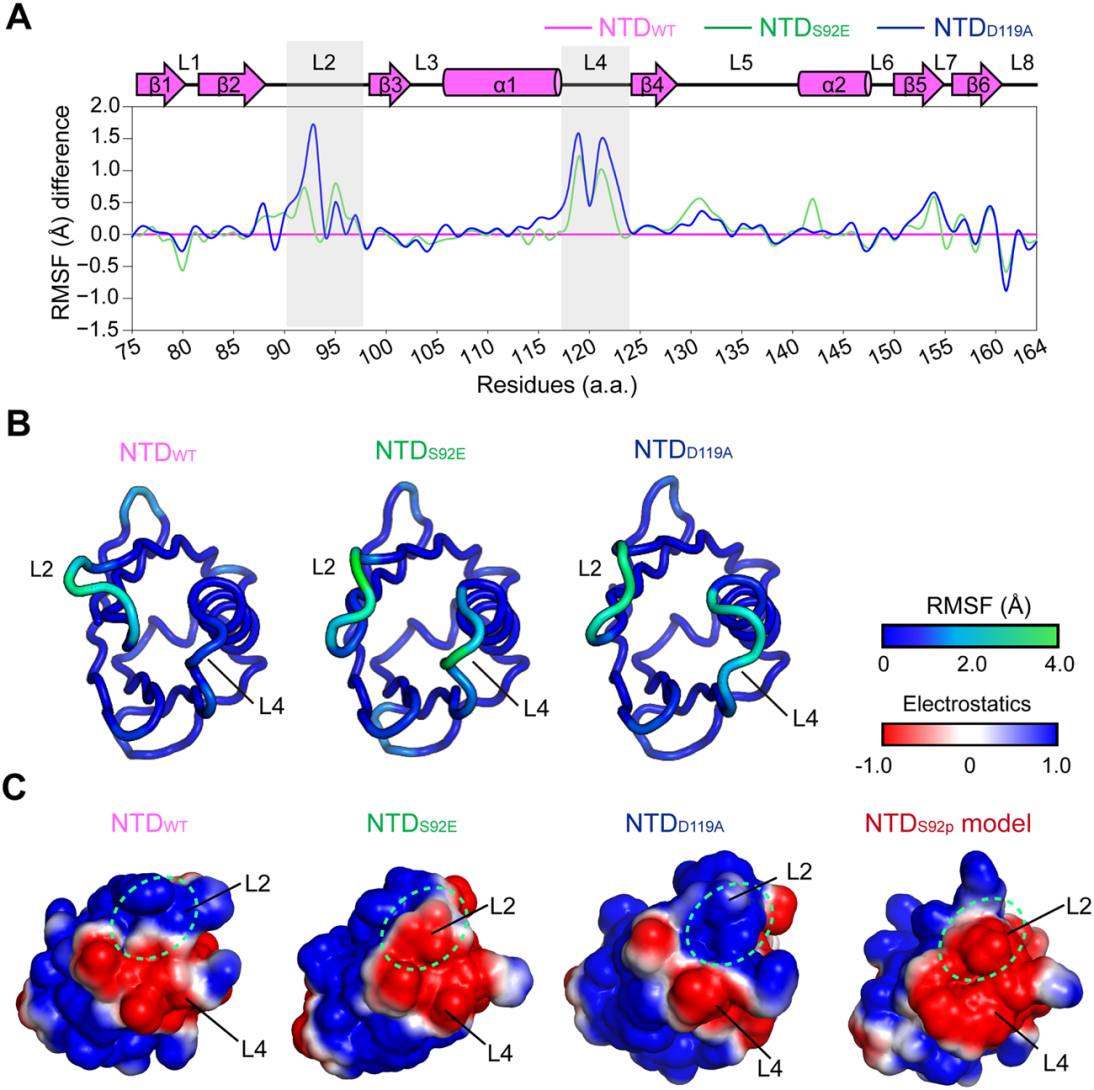
Structural dynamics and electrostatic surface charges in MCU NTD_WT_ and mutants. **(A)** Ensemble refinements of MCU NTD_WT_ (magenta; PDB ID: 4XSJ), MCU NTD_S92E_ (green), and MCU NTD_D119A_ (blue). Plots of the root-means-square fluctuation (RMSF) (Å) difference. The RMSF of each mutant, S92E (green) and D119A (blue), were relatively calculated by subtracting RMSF of the WT (magenta). Dramatic RMSF difference of the L2-L4 loops are highlighted in gray boxes. The β-strands (β1 − β6), α-helices (α1, α2), and loops (L1 − L8) are shown in arrows, cylinders, and lines, respectively. **(B)** Cartoon diagrams of ensemble refinements of MCU NTD_WT_, MCU NTD_S92E_, and MCU NTD_D119A_ in the RMSF (Å) coloured from blue (rigid) to green (flexible), depended on scoring of the residue dynamics. **(C)** Electrostatic surface charges of MCU NTD_WT_ and the mutants, S92E and D119A, and MD simulated MCU NTD_S92p_ model were calculated using PDB2PQR server with the CHARMM force field at the mitochondrial matrix pH of approximately 7.8, and the surface diagrams were generated using PyMOL software. The different surface charge regions are highlighted in green circles.

In addition, to understand whether the S92-D119 hydrogen bond disruption by S92p might contribute to flexibility of L2-L4 loops in the MCU NTD, we performed the ensemble refinement using PHENIX and calculated the root-mean-square fluctuation (RMSF) (Å) from the ensemble refinement results of the MCU NTD_WT_ and the mutants (S92E and D119A)^41,42^. Overall structures of two mutants showed similar RMSF scores in dynamics to that of the MCU NTD_WT_, while dramatic RMSF changes were observed in the L2-L4 loops of the MCU NTD mutants compared to the MCU NTD_WT_ (Fig. 3A,B).

To investigate whether phosphorylation of S92 in the MCU NTD affected electrostatic charges, we calculated and compared the side chain charges of the residues S92, S92p, S92E, and D119A, at the mitochondrial matrix pH of approximately 7.8 using the Henderson–Hasselbalch equation^43,44^. Negative charges in the mutant S92E (pKa ~4.3) and S92p (pKa_1_ ~1.5, pKa_2_ ~6.3) by deprotonation of the hydroxyl group were increased by −1.0 and −2.0, respectively, whereas the negative charge of −1.0 in the D119A mutant (pKa ~3.9) was reduced in comparison with the MCU NTD_WT_. In agreement with the changes of the negative charge, electrostatic surface charge was enhanced in the L2-L4 loops of the MCU NTD_S92E_ and the S92p model structures, whereas the positive surface charge of MCU NTD_D119A_ was increased compared to the MCU NTD_WT_ (Fig. 3C).

Collectively, these findings suggest that the S92-D119 hydrogen bond formation or disruption, which depends on S92 phosphorylation, regulates the conformation of L2-L4 loops and additional negative charges in the phosphate group of S92p in the MCU complex.

### Molecular dynamics simulation analysis of NTD_WT_ and NTD_S92p_ monomers

**Figure 4:**
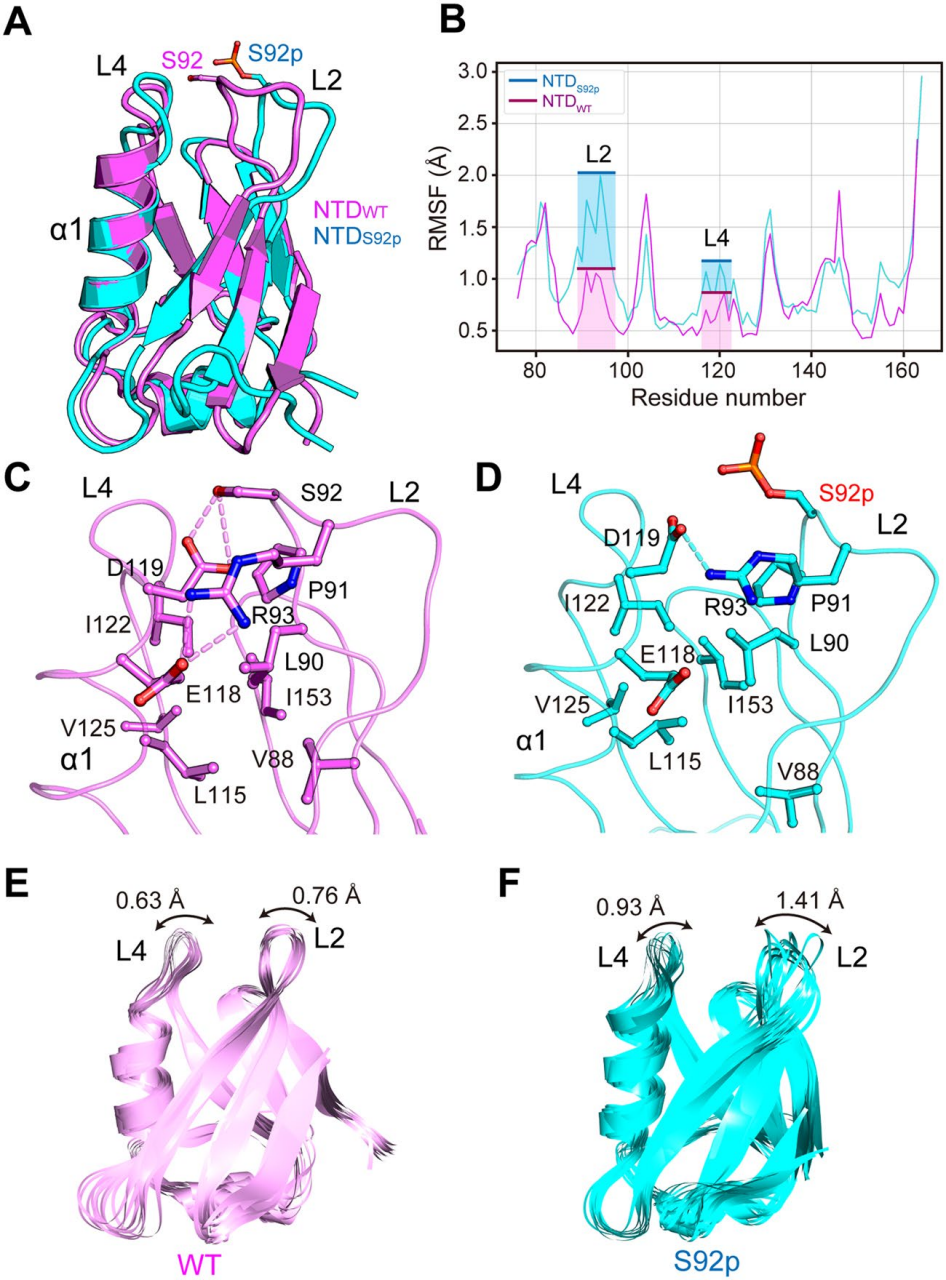
L2-L4 structure comparison between NTD_WT_ and NTD_S92p_ monomers during 10 ns molecular dynamics (MD) simulation. **(A-D)** MD snapshot structures of NTD_WT_ and NTD_S92p_ structures at 10 ns. **(A)** NTD_WT_ and NTD_S92p_ structures are superimposed and colored in pink and cyan, respectively. The S92 and S92p residues are displayed in a stick model with the same color. **(B)** The root-means-square fluctuation (RMSF) plots for C_α_ atoms of the NTD_WT_ and the NTD_S92p_ are shown as pink and cyan lines, respectively. The RMSF values of the L2 and L4 loops are highlighted by the boxes in the same color. **(C,D)** Atomic interaction analysis between residues of L2 and L4 loops on NTD_WT_ (pink) and NTD_S92p_ (cyan)_._ Hydrogen bond interactions are shown as dashed lines (pink in NTD_WT_ and cyan in NTD_S92p_). **(E,F)** MD snapshot structure superimposition. The 20 trajectories are extracted every 0.5 ns during 10 ns simulation time and aligned for NTD_WT_ (pink) (**E**) and NTDS_S92p._ (cyan) (**F**).

Molecular dynamics (MD) simulations were performed on the NTD_WT_ and NTD_S92p_ monomer structures to identify the intra structural changes caused by phosphorylation of S92 in the NTD. The MD simulations clearly showed the flexibility change of the L2-L4 loop region (Fig. 4A,E,F). The fluctuations of all amino acid residues in NTD_WT_ and NTD_S92p_ monomer structures were measured by plotting of the RMSF. The RMSF values of the L2 and L4 loops of the NTD_S92p_ structure were significantly higher than the values of the NTD_WT_ (Fig. 4B). The average RMSF values of the L2 loop for the NTD_WT_ and NTD_S92p_ were 0.76 Å and 1.41 Å, respectively; the values of the L4 loop were 0.63 Å and 0.93 Å, respectively.

To investigate details of the atomic interaction between the residues near the L2 and L4 loops, the final MD trajectory structure was extracted. In NTD_WT_, the S92 and R93 in the L2 loop were hydrogen-bonded with D119 in the L4 loop (Fig. 4C). Conversely, in NTD_S92p_, only R93 participated in the hydrogen bond interaction, as the interaction of S92 with D119 was broken (Fig. 4D). Therefore, it can be inferred that phosphorylation on S92 can break the interaction between the S92 and D119.

### Effects of S92 phosphorylation on the dimerization of the MCU-EMRE tetramer

**Figure 5:**
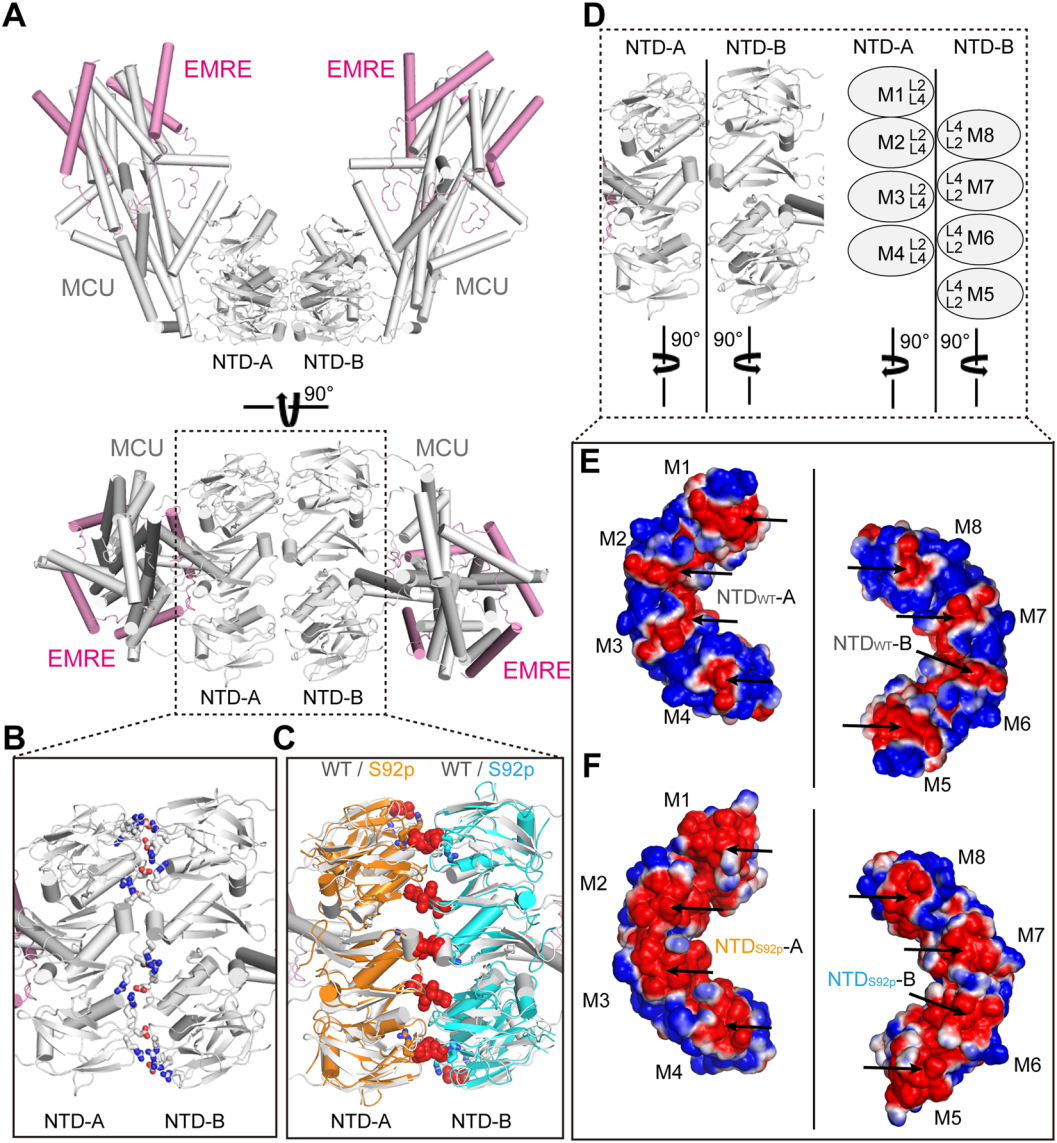
Structural comparison between the NTD_WT_ and the NTD S92-phosphorylated (NTD_S92p_) model in the dimerization of two MCU-EMRE channels. **(A)** The overall structure of two MCU-EMRE channels (PDB ID: 6O58). MCU and EMRE are shown in grey and magenta colored ribbons, respectively. **(B)** Detailed view of the interacting surfaces of NTD-A and NTD-B in dimer of the two MCU-EMRE channels. The residues (R93, D123, and R124) forming salt bridges and hydrogen bonds are shown in sticks. **(C)** Detailed view of the superimposed MCU NTD_S92p_ MD simulated model structures (10-nsec snapshot) onto NTD_WT_-A or -B of the two MCU-EMRE channel complexes. The MCU NTD_S92p_ residues that are expected to disrupt salt bridges and hydrogen bonds (R93, D123, and R124) are depicted as orange sticks and cyan sticks, respectively. Atomic clashes between NTD-A and NTD-B of the MCU NTD_S92p_ are denoted by red spheres. **(D‒F)** Differences between the electrostatic surface charges of the interacting interfaces of the MCU NTD_WT_ -A or -B (M1‒M8) superimposed on the MCU NTD_S92p_ -A or -B (M1‒M8). Enhancement of negative charges in the MCU NTD_S92p_ are highlighted with black arrows. Blue surfaces and red surfaces in the NTD_WT_ and in the MD simulated NTD_S92p_ indicate positive and negative charges, respectively.

Upon examination of the structures of the MCU NTD_S92E_, MCU NTD_D119A_, and MD simulated S92p models, we expected that S92 phosphorylation in the MCU induces conformational changes as well as enhancements in the negative charges in the local L2-L4 loops (Figs. 2 and 4). Wang *et al*. recently reported that the tetrameric MCU-EMRE channels underwent extensive interactions with each other resulting in the formation of dimers at the MCU NTDs, including the L2-L4 loops (Fig. 5A,B,D). Additionally, the MCU-EMRE channel interactions formed a V-shaped tetrameric MCU-EMRE dimer. Moreover, a single mutation, D123R, in the L4 loop of the MCU NTD abolished the dimerization of the two MCU-EMRE channels, possibly by disrupting the electrostatic interactions with the neighboring arginine residues, R93 and R124^19^. To elucidate the effect of S92p on the dimerization of tetrameric MCU-EMRE, we compared the binding energy difference for the dimerization of the tetramer between WT and S92p NTDs using the PRODIGY web server^45^. The binding free energy of NTD S92p (−7.4 kcal/mol) was higher than that of WT (−10.5 kcal/mol), suggesting that the conformational changes and enhancement of negative charges by S92 phosphorylation may affect the dimerization of two MCU-EMRE channels (Fig. 5C–F).

**Figure 6:**
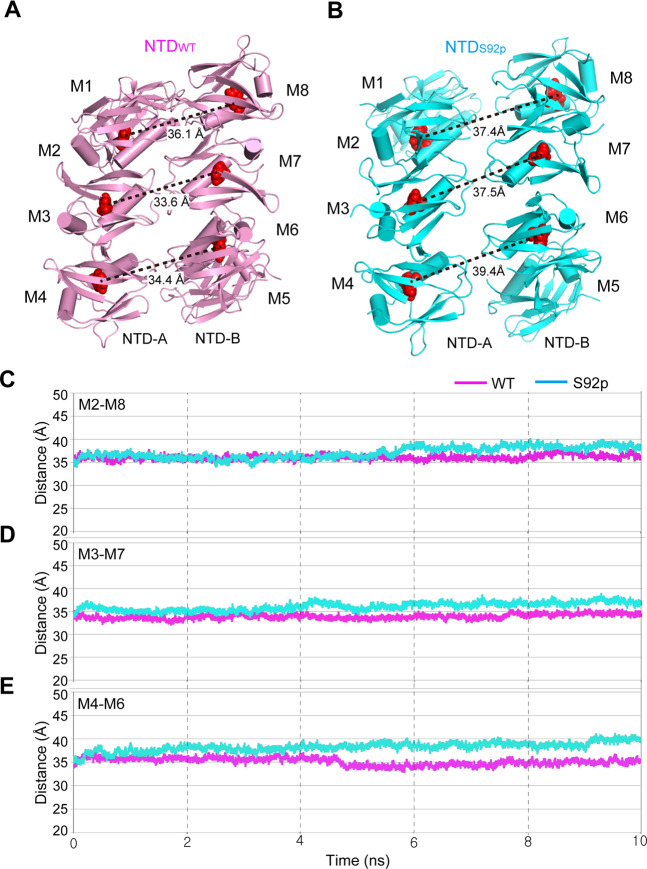
Distance comparison between NTD_WT_ and NTD_S92p_ octamers using MD simulation structures. **(A-B)** The 10 ns-snapshot structures of NTD_WT_ (**A**) and NTD_S92p_ octamers (**B**) are shown in pink and cyan, respectively. Each of the eight monomers are designated as M1 − M8. The distances between the center of monomer were calculated. **(C-E)** The three key monomer pair distances in the central region of the octamer were measured during 10 ns simulations times: M2-M8 (**C**), M3-M7 (**D**), and M4-M6 (**E**). The C_α_ atom of F111 (red sphere) located at the nearest position of the center of monomer was selected for the distance measurement.

To confirm this hypothesis, we performed MD simulations for the NTD_WT_ and NTD_S92p_ octamer structures. To compare the distance between the two tetramers (NTD-A and NTD-B), the three key monomer pair distances (M2-M8, M3-M7, and M4-M6) were monitored during 10 ns simulations times (Fig. 6). The snapshot structures at 10 ns (Fig. 6A,B) and the distance trajectory during the MD simulation (Fig. 6C–E) show that the distances between the paired monomers in NTD_S92p_ were significantly increased compared to that of the NTD_WT_ by approximately 1.5 to 5 Å. It suggests that the additional negative charges from the phosphate group might contribute to push each tetramers (NTD-A and NTD-B) away. Overall, our MD simulation studies suggest that the S92 phosphorylation can weaken dimerization of the MCU-EMRE tetramer.

## Discussion

MCU activity is modulated by its regulatory proteins, including MICU1, MICU2, MCUb, EMRE, and MCUR1, as well as post-translational modifications such as phosphorylation^2,7,8,20,21^. In addition, the MCU NTD plays pivotal roles in MCUR1 interaction, MCUb NTD interaction, Mg^2+^ binding selectivity, phosphorylation, redox sensor, oligomerization of MCU-EMRE channel complexes, and regulation of MCU Ca^2+^ uptake activity^6,7,17–19^. Thus, we believe functional roles of MCU NTD for its Ca^2+^ uptake activity warrants further investigation, although recent studies of NTD deletion of MCU appears to be functionally dispensable in mitochondrial Ca^2+^ uptake^19,46^

Protein kinases can be localized in the sarcoplasmic reticulum (SR) and mitochondria, and modulate function of Ca^2+^ channels by phosphorylation. Phosphorylation of Ca^2+^ channels containing ryanodine receptor 2 and inositol 1,4,5-trisphosphate receptors regulates Ca^2+^ release in the SR through PKA and CaMKII^20,47–51^. The MCU is directly phosphorylated by Pyk2 and AMPK and phosphorylated MCU facilitates Ca^2+^ entry into the mitochondria^20,21^. The conserved S92 in the MCU is a putative recognition site (89-RLPS-92; RxxS motif) for phosphorylation by Ser/Thr kinases such as CaMKII, PKA, and PKC on the basis of prediction of KinasePhos 2.0 server and GPS 2.0 software^31–35^. Nguyen *et al*. observed S92 phosphorylation of MCU by CaMKII using MCU S92 phospho-specific antibody *in vivo*^25^, although the regulatory functions of MCU activity by CaMKII still remain controversial^22–26^. Instead, our *in vitro* kinase assay results indicated MCU S92 was phosphorylated by PKC isoforms (PKC_βII_, PKC_δ_, and PKC_ε_) localized in the mitochondrial matrix, but was not phosphorylated by PKA (Fig. 1D,F)^27,28^. Further studies are needed to understand the functional roles of MCU NTD phosphorylation by PKC isoforms.

Free radicals, such as ROS and reactive nitrogen species (RNS), generate in a well-modulated manner to maintain cellular homeostasis as signalling second messengers, and play critical roles in the activation of enzymes and alteration of lipids, protein, and DNA^52,53^. Under physiological conditions, the MCU uptakes Ca^2+^ ions into the matrix; Ca^2+^ ions play an essential role modulating ATP synthesis through TCA cycle and the electron transfer chain, and finally induce ROS production as by-products in the mitochondria^54^. However, continuous overload of mitochondrial Ca^2+^ entry can produce large amounts of ROS and eventually lead to apoptotic or necrotic cell death^54^. Upon production of ROS, PKC_βII_, PKC_δ_, and PKC_ε_ translocate to the mitochondria and modulate functions of enzymes and Ca^2+^ channels by Ser/Thr phosphorylation, as well as ROS production^27–29^. In our studies, we observed that PKC_βII_, PKC_δ_, and PKC_ε_ phosphorylated the MCU_S92_ *in vitro*. We speculate that regulation of the S92 phosphorylation by the PKC isoforms under physiological conditions play important roles in ROS homeostasis or programmed cell death by excessive ROS, driven by the MCU Ca^2+^ uptake. Additional experimental evidence will be required to clarify the functional roles of PKC isoforms in the MCU^29–31^.

In conclusion, we identified that the PKC isoforms, PKC_βII_, PKC_δ_, and PKC_ε_, are capable of phosphorylating S92 in the MCU NTD. We also characterized local conformational changes in our structural determination of MCU NTD_S92E_ and NTD_D119A_ as well as in MD simulation analysis of the WT and S92p. The conformational changes and enhancement of negative charge of the L2-L4 loops in the MCU NTD by S92 phosphorylation may be essential for regulating MCU activity, despite there lacks of functional data for the MCU activity modulation by S92 phosphorylation. Further studies are required to reveal the functional effects of MCU S92 phosphorylation by the PKC. The results provide a framework for further studies investigating the functional and structural roles of MCU phosphorylation by PKC.

## Materials and Methods

### DNA constructs

For structural studies, human MCU NTD (residues 75 − 165), including N-terminal His_6_-bacteriophage T4 lysozyme (residues 2 − 161; triple mutants of D20N/C54T/C97A)^55,56^ in the modified pET21a vector (Novagen), was constructed as previously described^6^. Point mutagenesis using polymerase chain reaction (PCR) was performed to construct the S92E or D119A mutants. For the *in vitro* kinase assay of the MCU NTD S92, we designed all Ser/Thr to Ala mutants (T76, S87, S92, T100, S105, S107, S129, S138, T139, and T157; MCU NTD^AA^). PCR was used to synthesize the MCU NTD^AA^ construct using 12 oligonucleotides; then, MCU NTD^AA^_S92_ was generated using the A92S mutation from the MCU NTD^AA^.

### Purification of MCU constructs

The T4 lysozyme-MCU NTD_S92E_ or T4 lysozyme-MCU NTD_D119A_ was purified using the same method for T4 lysozyme-MCU NTD_WT_ as previously described^6^. The proteins were expressed in the *Escherichia coli* strain BL21-CodonPlus (DE3), followed by purification using Ni-NTA affinity and size exclusion chromatography (SEC) on a HiLoad 16/60 Superdex 75 column (GE Healthcare Life Science). The samples were concentrated by centrifugation using Amicon Ultra-15 10 K filter units (Millipore) to 5 mg mL^−1^ in final buffer containing 20 mM Tris-HCl (pH 8.0), 50 mM NaCl, 5% (*v*/*v*) glycerol, and 1 mM DTT.

For *in vitro* kinase assays, MCU NTD_WT_ and MCU NTD^AA^_S92_ were purified by a similar procedure using the following buffers: lysis buffer [50 mM Tris-HCl (pH 8.0), 500 mM NaCl, 10 mM imidazole, 5% (*v*/*v*) glycerol, 1 mM PMSF, 1 mM β-mercaptoethanol], wash buffer [50 mM Tris-HCl (pH 8.0), 500 mM NaCl, 40 mM imidazole, 5% (*v*/*v*) glycerol], and elution buffer [50 mM Tris-HCl (pH 8.0), 500 mM NaCl, 500 mM imidazole, 5% (*v*/*v*) glycerol]. The samples were then purified using SEC on a HiLoad 16/60 Superdex 75 column (GE Healthcare Life Science) pre-equilibrated with a buffer [50 mM Tris-HCl (pH 7.5) and 150 mM NaCl]. Then, the fractions containing human MCU NTD_WT_ and MCU NTD^AA^_S92_ were collected. The protein was concentrated using an Amicon Ultra-15 10 K filter unit (Millipore) at a concentration of 0.3 mg mL^−1^. Final human MCU NTD proteins were stored at −80 °C.

### *In vitro* kinase assays

Six micrograms (6 μg) of purified MCU NTD_WT_, 20 μg of purified MCU NTD^AA^_S92_, and 32 μg of commercially obtained MBP (Enzo, ALX-202-075) were phosphorylated by PKA (Promega, V5161), PKC mixtures (α, β, and γ isoforms with lesser δ and ζ; Promega, V5261), PKC_βII_ (Promega, V3741), PKC_δ_ (Promega, V3401), and PKC_ε_ (Promega, V4036). *In vitro* phosphorylation of PKA was performed in 25 mM Tris-HCl (pH 7.5), 10 mM MgCl_2_, 2 mM DTT, 5 mM β-glycerophosphate, 0.1 mM Na_3_VO_4_, 0.2 mM Mg-ATP, and 3 pmol of [γ-^32^P]ATP (3000 Ci/mmol) with 20 ng PKA for MBP and 100 ng PKA for MCU NTD_WT_ and MCU NTD^AA^_S92_. *In vitro* phosphorylation assays of PKC mixtures, PKC_βII_, PKC_δ_, and PKC_ε_ were performed in 1 × reaction buffer A (SignalChem, K03-09) [20 mM Tris-HCl (pH 7.5), 10 mM MgCl_2_, 0.02% (*v*/*v*) Tween-20], 2 mM DTT (SignalChem, D86-09B), 1 × PKC lipid activator (SignalChem, L51-39), 0.2 mM Mg-ATP, and 3 pmol of [γ-^32^P]ATP (3000 Ci/mmol) with 100 ng PKC mixtures, PKC_βII_, PKC_δ_, and PKC_ε_ for MBP and 100 ng PKC, 50 ng PKC_βII_, 200 ng PKC_δ_, and 200 ng PKC_ε_ for MCU NTD_WT_, and MCU NTD^AA^_S92_. All *in vitro* kinase assays were performed at 30 °C for 60 min. The reaction was halted by the addition of SDS-PAGE sample buffer. Then, reaction samples were resolved by SDS-PAGE and visualized by autoradiography.

### Crystallization

The T4 lysozyme-MCU NTD_S92E_ or T4 lysozyme-MCU NTD_D119A_ was crystalized using the same method for T4 lysozyme-MCU NTD_S92A_ as previously described^6^. Crystals of T4 lysozyme-MCU NTD_S92E_ or T4 lysozyme-MCU NTD_D119A_ were produced using the hanging drop vapour diffusion and microseeding method, using T4 lysozyme-MCU NTD_WT_ crystals as seeds in the reservoir solution containing 20% (*w/v*) polyethylene glycol (PEG) 3350, 5% (*v*/*v*) glycerol, 0.3 M (NH_4_)_2_SO_4_, and 0.1 M Bis-Tris-HCl (pH 5.5). Once the microcrystals (<0.01 − 0.02 mm) of the T4 lysozyme-MCU NTD_WT_ grew at 20 °C, 2 μL of T4 lysozyme-MCU NTD_S92E_ or T4 lysozyme-MCU NTD_D119A_ proteins and 2 μL of the reservoir solution were added directly to the 1 μL drop containing T4 lysozyme-MCU NTD_WT_ seed crystals. The final T4 lysozyme-MCU NTD_S92E_ or NTD_D119A_ crystals were grown at 20 °C in 5 μL mixtures containing the WT and the S92E or D119A mutant at a 1:4 molar ratio. The crystals were directly flash-frozen in liquid nitrogen.

### Data collection, structure determination, and refinement

Diffraction data of T4 lysozyme-MCU NTD_S92E_ or T4 lysozyme-MCU NTD_D119A_ crystals were collected at 100 K using synchrotron X-ray sources on beamlines 5 C at the Pohang Acceleratory Laboratory (PAL) (Pohang, South Korea). We finally collected diffraction data for T4 lysozyme-MCU NTD_S92E_ at a resolution of 2.50 Å and for T4 lysozyme-MCU NTD_D119A_ at 2.85 Å using a single wavelength, 0.9795 Å. The diffraction data were processed using the HKL2000 suite^57^. Molecular replacement was carried out using Phaser in the CCP4 suite^58^, using the structures of the bacteriophage T4 lysozyme (PDB ID: 2LZM) and MCU NTD (PDB ID: 4XTB) as templates. The obtained models were subjected to iterative rounds of model building and refinement using programs Coot^59^ and REFMAC5 in CCP4 suite^58^. The details of data collection and refinement statistics are provided in Table 1.

### Structural analysis

All structural figures were generated using PyMOL version 1.5.0.4 (Schrödinger LLC). The amino acid sequence and protein surface conservation of the MCU NTD were calculated using the ConSurf server^30^. The CCP4 program LSQKAB was used to superimpose the structures of MCU NTD_WT_ (PDB ID: 4XTB), MCU NTD_S92E_, and MCU NTD_D119A_ and to estimate RMSD (Å) scores of C_α_ atoms^58^. The electrostatic surface charges of MCU NTDs (WT, S92E, S92p, and D119A) were analysed using the PDB2PQR server^60^ and visualized using PyMOL version 1.5.0.4 (Schrödinger LLC).

### Ensemble refinement

Ensemble refinement for T4 lysozyme-MCU NTD_WT_ (PDB ID: 4XSJ), T4 lysozyme-MCU NTD_S92E_, and T4 lysozyme-MCU NTD_D119A_ was performed using structures and structural factors by phenix.ensemble_refinement^41^. Default parameters were used in the phenix.ensemble_refinement, including *pTLS* = 0.8 and *T*_*bath*_ = 5 K, and solvent updated every 25 cycles. The simulations have an equilibration phase (10τx) in which the temperature, X-ray weight and averaged structure factors stabilize, followed by an acquisition phase (10τx). The output structures by ensemble refinement were visualized using PyMOL version 1.5.0.4 (Schrödinger LLC) with a script ‘ens_tool.py’. The RMSF difference histogram for the MCU NTD_WT_ and mutants (S92E and D119A) was plotted using SigmaPlot 12.

### Molecular dynamic simulations

Four molecular dynamics (MD) simulations of NTD_WT_ and NTD_S92p_ monomer and octamer structures were carried out using GROMACS (GROningen Machine for Chemical Simulations) 2018.4 package^61–63^ with amber99sb-star-ILDNP force field^64^. Molecular topologies for phosphorylated S92 were generated by AnteChamber Python Parser interface (ACPYPE) with generalized AMBER force field 2 (GAFF2)^65,66^. All four systems were solvated with TIP3P water molecules^67^ in a dodecahedron box and Na^+^ counter ions were added to neutralize the net changes of the systems by replacing water molecules. In all cases, bond lengths were constrained with LINCS^68^ and long-range electrostatics were calculated using the smooth particle mesh Ewald (PME) method with a cut-off of 1.0 nm^69,70^. A cut-off of short-range non-bonded interactions, van der Waals (vdW), were truncated at 1.0 nm. All MD simulations were conducted energy minimization using the steepest descent method. Equilibration was then performed in two phases, during which position restraints applied to all heavy atoms of the protein. First, the simulations were run under NVT conditions at 300 K, using Berendsen’s coupling algorithm^71^ for 100 ps. The second phase of equilibration was carried out an NPT ensemble for 100 ps, using the Nose-Hoover thermostat^72,73^ and the Parrinello-Rahman barostat^74,75^ with coupling time constants of 2.0 ps and 5.0 ps to maintain 300 K and 1 bar, respectively. Production MD was then conducted for 10 ns without any restraint and under the same conditions as the NPT ensemble. All analyses of MD simulation results were performed using the analysis tools in the GROMACS package.

### Accession numbers

Atomic coordinates and structure factors of T4 lysozyme-MCU NTD_S92E_ and T4 lysozyme-MCU NTD_D119A_ have been deposited in the PDB with the accession numbers, 6JG0 and 6KVX, respectively.

## Supplementary information

Supplementary information.
