# Supplementary material for: S92 phosphorylation induces structural changes in the N-terminus domain of human mitochondrial calcium uniporter
Source: Sci Rep. 2020 Jun 4;10:9131. doi: 10.1038/s41598-020-65994-y (PMC7272466; doi:10.1038/s41598-020-65994-y)
Supplement: Supplementary file 1 — Supplementary information. [file 41598_2020_65994_MOESM1_ESM.pdf]

Supplementary Information

S92 phosphorylation induces structural changes in the N-terminus domain of human mitochondrial calcium uniporter

Youngjin Lee[1,2,4†*], Jongseo Park[1,2†], Gihwan Lee[5], Sanghwa Yoon[5], Choon Kee Min[1,3], Tae Gyun Kim[1,2,§], Takenori Yamamoto[6,7], Do Han Kim[1,3], Keun Woo Lee[5], and Soo Hyun Eom[1,2*]

[1]School of Life Sciences, [2]Steitz Center for Structural Biology, [3]Systems Biology Research Center, Gwangju Institute of Science and Technology (GIST), Buk-gu, Gwangju 61005, Republic of Korea. [4]Infection and Immunity Research Laboratory, Metabolic Regulation Research Center, Korea Research Institute of Bioscience and Biotechnology (KRIBB), Daejeon 34141, Republic of Korea. [5]Division of Life Science, Division of Applied Life Science (BK21 Plus), Plant Molecular Biology and Biotechnology Research Center (PMBBRC), Research Institute of Natural Science (RINS), Gyeongsang National University (GNU), 501 Jinju-daero, Jinju, 52828, Republic of Korea. [6]Institute for Genome Research, Tokushima University, Kuramotocho-3, Tokushima 770-8503, Japan. [7]Faculty of Pharmaceutical Sciences, Tokushima University, Shomachi-1, Tokushima 770-8505, Japan.

[†]These authors contributed equally to this work.

*Corresponding authors:

Youngjin Lee: yjlee86@kribb.re.kr (Phone: +82-42-879-8222, Fax: +82-42-879-8595)

Soo Hyun Eom: eom@gist.ac.kr (Phone: +82-62-715-2493, Fax: +82-62-715-2521)

Experimental methods

Supplementary Figure Legends

Supplementary Fig. 1. Amino acid sequence conservation of N-terminal domain of human mitochondrial calcium uniporter (MCU). Amino acid sequence of the MCU NTD coloured by scoring the residue conservation from 230 MCU NTD homologues using the ConSurf sever. The amino acid sequence 89-RLPS-92 (RxxS motif), which is a putative recognition site for serine/threonine kinases, is highlighted by a black underline. The S92 residue is highlighted by the red triangle.

Supplementary Fig. 2. *In vitro* kinase assays of myelin basic protein. Autoradiography analysis of myelin basic protein (MBP) that were incubated with [γ-^{32}P]ATP (P-32), protein kinase A (PKA), or protein kinase C (PKC) isoforms (α, β, γ mixtures, $PKC_{\beta II}$, PKC_{δ}, and PKC_{ε}). The reaction samples were resolved by SDS-PAGE, and visualized by autoradiography. Full autoradiography results in Supplementary Fig S4. Data are representative of three independent experiments.

Supplementary Fig. 3. Structural comparison between the MCU NTD_{S92A} and NTD_{WT} or NTD_{S92E}. (A,B) Detailed view of superimposed L2-L4 loops of MCU NTD_{WT} (PDB ID: 4XSJ, magenta) and MCU NTD_{S92A} (PDB ID: 5BZ6, orange) (A) or MCU NTD_{S92A} and MCU NTD $_{S92E}$ (green) (B). The backbones and side chains are represented ribbons and sticks, respectively. The arrows represent movement of the residues participating in hydrophobic interactions (black) or hydrogen bonds (red). Dashed-lines (magenta in WT, orange in S92A, and green in S92E) denote hydrogen bonds.

Supplementary Fig. 4. Full length autoradiography results in Figs. 1D–1F and Supplementary Fig. 2. Red lines show the cropping locations.

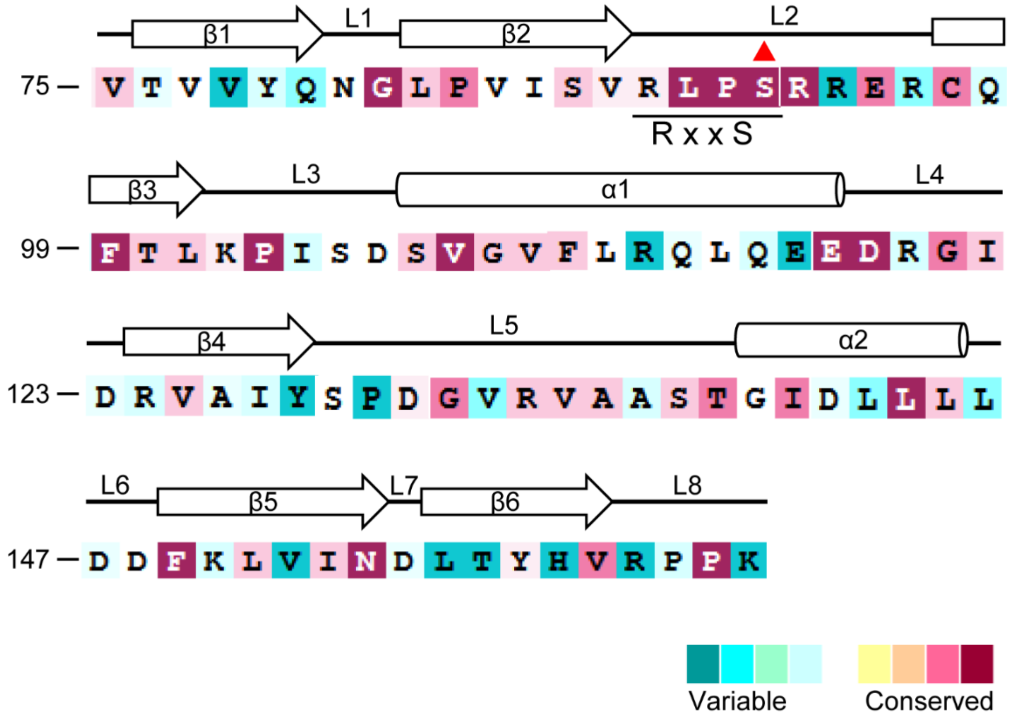

Supplementary Fig. 1.

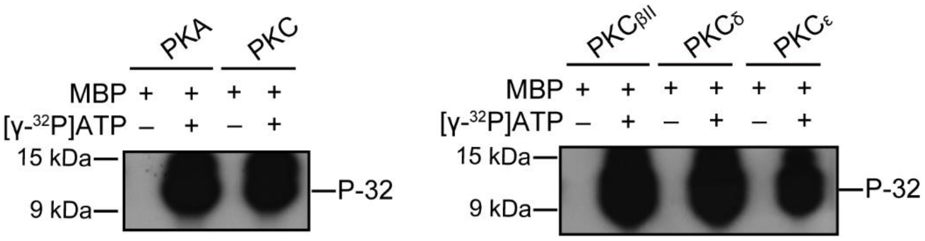

Supplementary Fig. 2.

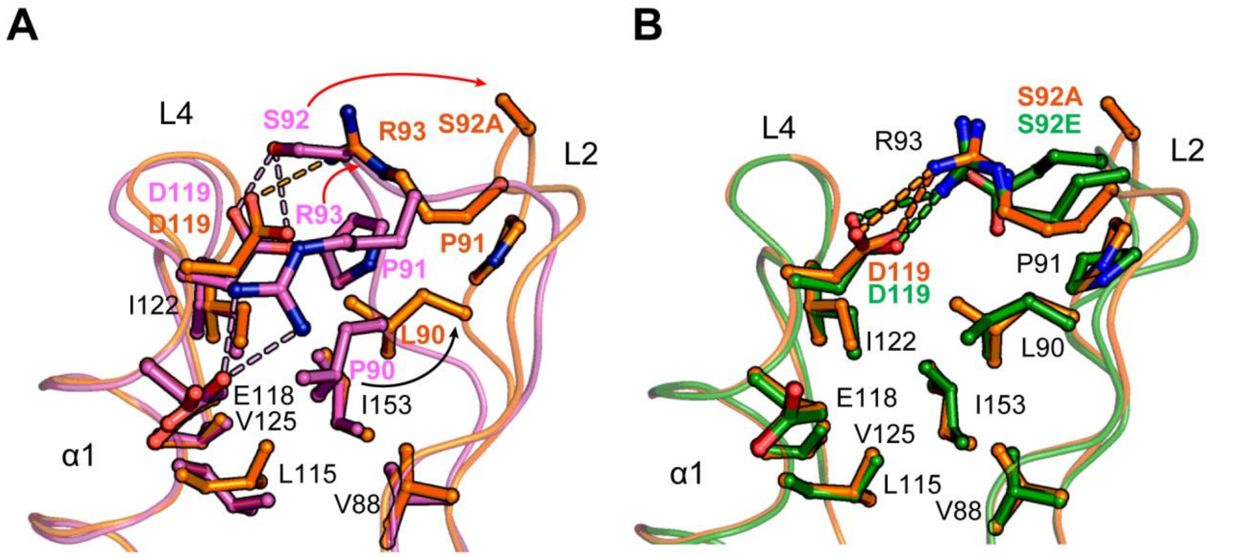

Supplementary Fig. 3.

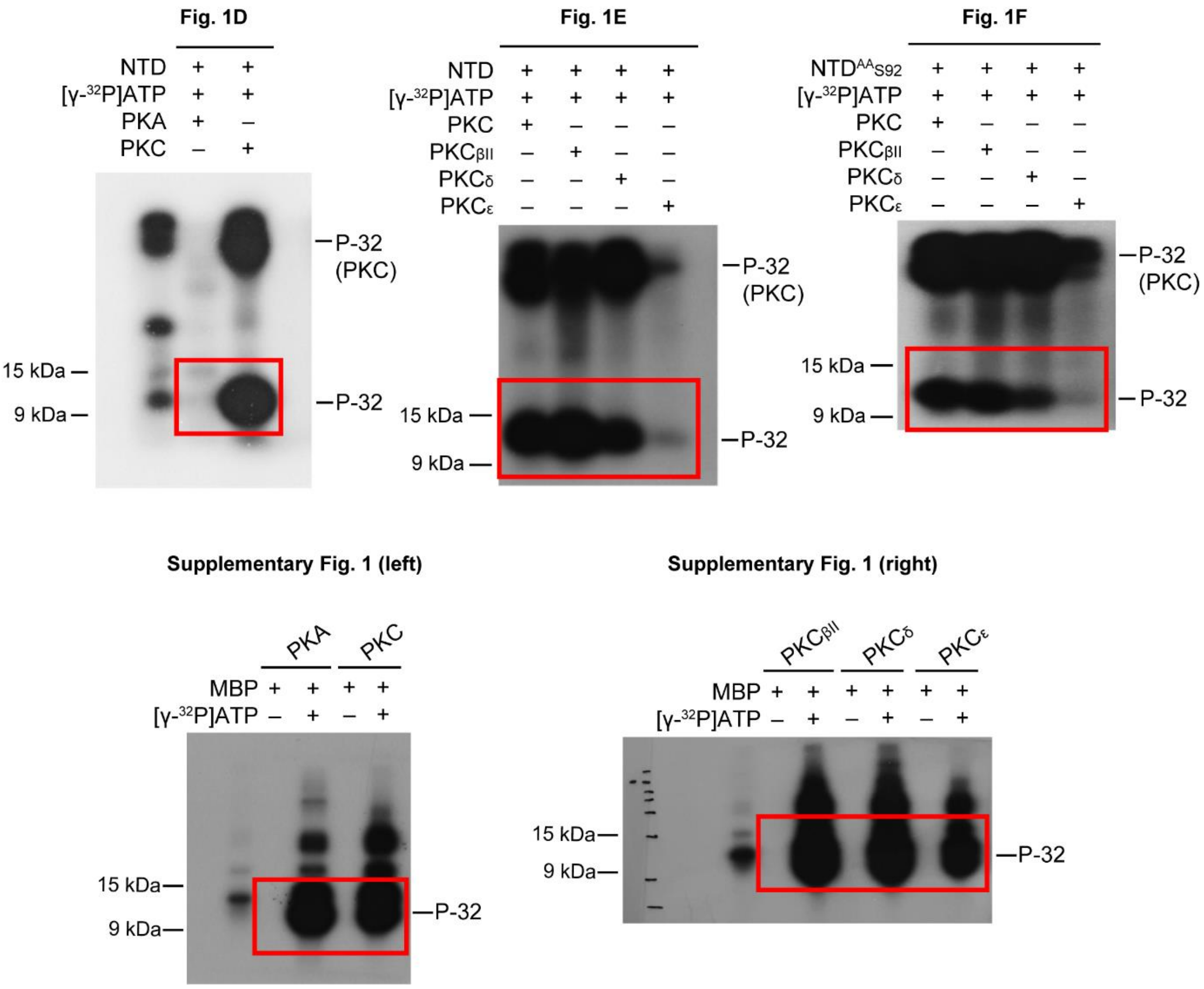

Supplementary Fig. 4.
